# Cannabinoid CB2 Receptor Potentiates Obesity-Associated Inflammation, Insulin Resistance and Hepatic Steatosis

**DOI:** 10.1371/journal.pone.0005844

**Published:** 2009-06-09

**Authors:** Vanessa Deveaux, Thomas Cadoudal, Yasukatsu Ichigotani, Fatima Teixeira-Clerc, Alexandre Louvet, Sylvie Manin, Jeanne Tran-Van Nhieu, Marie Pierre Belot, Andreas Zimmer, Patrick Even, Patrice D. Cani, Claude Knauf, Remy Burcelin, Adeline Bertola, Yannick Le Marchand-Brustel, Philippe Gual, Ariane Mallat, Sophie Lotersztajn

**Affiliations:** 1 INSERM, U955, Créteil, France; 2 Université Paris Est, Faculté de Médecine, Créteil, France; 3 AP-HP, Groupe Hospitalier Henri Mondor – Albert Chenevier, Service d'Hépatologie et de Gastroentérologie, Créteil, France; 4 AP-HP, Groupe Hospitalier Henri Mondor – Albert Chenevier, Département de Pathologie, Créteil, France; 5 Department of Molecular Psychiatry, University of Bonn, Bonn, Germany; 6 INRA, UMR914, AgroParisTech, Physiologie de la Nutrition et du Comportement Alimentaire, CNRH-IdF, Paris, France; 7 INSERM, U858, Toulouse, France; 8 INSERM, U895, Team 8, Nice, France; 9 University of Nice-Sophia-Antipolis, Faculty of Medicine, Nice, France; 10 Centre Hospitalier Universitaire of Nice, Digestive Center, Nice, France; University of Camerino, Italy

## Abstract

**Background:**

Obesity-associated inflammation is of critical importance in the development of insulin resistance and non-alcoholic fatty liver disease. Since the cannabinoid receptor CB2 regulates innate immunity, the aim of the present study was to investigate its role in obesity-induced inflammation, insulin resistance and fatty liver.

**Methodology:**

Murine obesity models included genetically leptin-deficient *ob/ob* mice and wild type (WT) mice fed a high fat diet (HFD), that were compared to their lean counterparts. Animals were treated with pharmacological modulators of CB2 receptors. Experiments were also performed in mice knock-out for CB2 receptors (*Cnr2* −/−).

**Principal Findings:**

In both HFD-fed WT mice and *ob/ob* mice, *Cnr2* expression underwent a marked induction in the stromal vascular fraction of epididymal adipose tissue that correlated with increased fat inflammation. Treatment with the CB2 agonist JWH-133 potentiated adipose tissue inflammation in HFD-fed WT mice. Moreover, cultured fat pads isolated from *ob/ob* mice displayed increased *Tnf* and *Ccl2* expression upon exposure to JWH-133. In keeping, genetic or pharmacological inactivation of CB2 receptors decreased adipose tissue macrophage infiltration associated with obesity, and reduced inductions of *Tnf* and *Ccl2* expressions. In the liver of obese mice, *Cnr2* mRNA was only weakly induced, and CB2 receptors moderately contributed to liver inflammation. HFD-induced insulin resistance increased in response to JWH-133 and reduced in *Cnr2* −/− mice. Finally, HFD-induced hepatic steatosis was enhanced in WT mice treated with JWH-133 and blunted in *Cnr2* −/− mice.

**Conclusion/Significance:**

These data unravel a previously unrecognized contribution of CB2 receptors to obesity-associated inflammation, insulin resistance and non-alcoholic fatty liver disease, and suggest that CB2 receptor antagonists may open a new therapeutic approach for the management of obesity-associated metabolic disorders.

## Introduction

Along with the alarming rise in obesity to epidemic proportions, related complications such as diabetes mellitus, insulin resistance, cardiovascular disease and non-alcoholic fatty liver disease (NAFLD) are also growing and represent some of the leading health-care concerns [1]–[3]. Several studies have conclusively demonstrated that a chronic low-grade systemic inflammatory state, predominantly arising from adipose tissue and liver, plays a critical role in the development of these obesity-related comorbidities [4]–[7]. Thus, in adipose tissue, fat laden adipocytes initiate the inflammatory response by producing cytokines and chemokines, including MCP-1, TNFα and osteopontin, that promote recruitment of proinflammatory macrophages and further amplification of local inflammation [4]–[9]. Increased adipose tissue inflammation will contribute to insulin resistance by disrupting insulin-dependent signaling pathways, leading to increased delivery of free fatty acids to the liver, and thereby contributing to hepatic steatosis [10]. Moreover, fatty liver itself also induces a local subacute inflammatory state characterized by production of inflammatory mediators such as TNFα or IL-6, that directly contribute to hepatic and systemic insulin resistance [3].

Cannabinoid receptors CB1 and CB2 belong to the family of G protein-coupled receptors, and bind exogenous ligands derived from *Cannabis Sativa* as well as endogenous arachidonic-derived ligands (endocannabinoids) among which anandamide or 2-arachidonoylglycerol are the best characterized [11]–[13]. CB1 receptors play a key role in the pathogenesis of obesity, insulin resistance and NAFLD *via* central and peripheral effects [11], [14]. CB2 receptors are primarily expressed in cells of the immune system, including macrophages, and regulate the inflammatory response in various settings [15]. Thus, preclinical studies have demonstrated the critical role of CB2 receptors in the inflammatory process associated with rheumatoid arthritis, inflammatory bowel diseases, atherosclerosis or liver ischemia-reperfusion injury [15]–[18]. The aim of the present study was therefore to investigate the role of CB2 receptors in obesity-associated inflammation, insulin resistance and NAFLD.

## Methods

### Animals and Experimental Design

Mice used for the experiments included wild type (WT) C57Bl/6J, *ob/ob* and *ob+/ob* male mice (Janvier, France) and mice with a targeted mutation of the *Cnr2* gene [19]. To obtain single mutant mice on an inbred congenic genetic background, heterozygous *Cnr2* +/− mice were backcrossed with WT C57Bl/6J (The Jackson Laboratory) animals over 10 generations. Heterozygous mice from the N10 generation were intercrossed to homozygous *Cnr2* −/− animals. Genotyping was performed on tail genomic DNA using QuantiTect™ SYBR® Green PCR kit (Qiagen), with the following primers: mouse *Cnr2*: sense 5′-CTGTGCTGCTCATATGCTGG-3′, antisense 5′-GCAGAGCGAATCTCTCCACT-3′. We also verified that *Cnr2* −/− mice still expressed CB1 receptors, using the following primers: mouse *Cnr1*: sense 5′-CTCCTGGCACCTCTTTCTCAG-3′, antisense 5′-GTCTCCTGCTGGAACCAACGG-3′.

Experimental protocols were conducted in accordance with French government policies (Services véterinaires de la Santé et de la production animale, Ministère français de l′Agriculture). Adult male mice were housed under 12 hours of light/12 hours of dark cycle, in a temperature-controlled environment. WT C57Bl/6J mice and *Cnr2* −/− mice (7–10 week-old) were fed a standard chow (TD 2016, Harlan) or a high fat diet (36% fat, TD 99249, Harlan) for 6 or 15 weeks, as indicated. Body weight and food intake were measured weekly.

The impact of the CB2 agonist JWH-133 (Tocris, France) was evaluated in 8-week-old C57Bl/6J mice under ND or HFD for 6 weeks that were treated with JWH-133 during the last 15 days. In some experiments, the effect of the CB2 antagonist AM-630 (Tocris, France) was evaluated in 6-week-old obese male mice (*ob/ob*) and lean counterparts (*ob+/ob*). JWH-133 and AM-630 were freshly dissolved in a vehicle solution containing 1 drop of Tween 20 in 0.1 ml dimethylsulfoxide (DMSO), sonicated, and further diluted 50 times in NaCl 9‰. Mice received a 15-day course of daily JWH-133 (3 mg/kg) intraperitoneal injection (n = 10 HFD-fed WT mice, and n = 6 ND-fed WT mice) or of 1 mg/kg AM-630 (n = 10 *ob/ob* and n = 5 *ob+/ob*). Control animals were treated in parallel with vehicle. Body weight and food intake were measured daily.

In all the experiments, mice were sacrificed after overnight fasting. White epididymal adipose tissue and liver were removed, weighed and either fixed in buffered formalin, or snap frozen in liquid nitrogen. All samples were stored at −80°C until use.

### Preparation of adipocyte and stromal vascular fraction (SVF) from adipose tissue

Epididymal adipose tissues from *ob+/ob* and *ob/ob* mice were chopped and rinsed in buffer A containing 120 mM NaCl, 4 mM KH_2_PO_4_, 1 mM MgSO_4_, 750 µM CaCl_2_, 10 mM NaHCO_3_ and 30 mM HEPES pH 7.4. Explants were incubated at 37°C for 30 minutes in 10 ml of buffer A supplemented with 1% BSA, 280 mM glucose and 15 mg of type 1 collagenase (Clostrido peptidase A, Sigma). Adipocytes were then collected by filtration and floatation, and the SVF was obtained following further centrifugation for 15 min at 260 g and two washings in buffer A. Purity of the fractions was evaluated by *Emr1* and *Adipoq* expressions (not shown). All samples were stored at −80°C until use.

### Preparation of hepatocyte and non parenchymal liver cell fraction

Livers from *ob+/ob* and *ob/ob* mice were perfused *in vivo* by Liberase® (Roche) through portal vein. Livers were then dissociated with forceps in Williams medium contanining 10% FCS. Hepatocytes were obtained after filtration and sedimentation while the supernatant was centrifugated at 300 g for 10 min to obtain the non parenchymal cell fraction.

### Adipose tissue and liver histology

Fat and liver specimens were fixed in 10% formalin, paraffin-embedded and tissue sections (8 and 4 µm, respectively) were stained with hematoxylin-eosin for routine examination. Adipocyte size was quantified on at least 30 cells from 3 separate fields in 7 WT and 5 *Cnr2* −/− HFD-fed animals, using Image J software. Hepatic steatosis was blindly assessed on 4 random fragments from different areas of each liver (n = 10 WT and n = 10 *Cnr2* −/− HFD-fed animals), and was staged on a scale of 0 to 4, according to the percentage of hepatocytes containing cytoplasmic vacuoles as follows: 0, (<5%), 1 (5–20%), 2 (20%–30%), 3 (30–60%), 4 (≥60%).

### Indirect calorimetry

*In vivo* indirect open circuit calorimetry was performed as previously described [20], [21]. Briefly, the mice (HFD-fed WT (n = 7) and HFD-fed *Cnr2−*/− (n = 7)) were housed individually in the metabolic cage at 09:30 with water, but without food. Temperature in the metabolic cage was maintained at 32°C±0.5 (low range of thermoneutrality), with an artificial 12 h–12 h light-dark cycle. Respiratory exchanges and spontaneous activity were continuously recorded at 10 s interval from 10:00 until 09:00 the following day. Resting and activity-related metabolic rates were calculated a posteriori using the Kalman filtering method [21]. Basal metabolic rate and basal RQ were defined as the mean resting metabolic rate measured between 16:00 and 18:00 when the mice were in a post-absorptive state, just before the 1g-test meal was given. Post meal RQ was defined as the mean RQ measured during 6 hrs following administration of the 1g-test meal (until metabolic rate returned to pre-meal values); peak post meal is defined as the highest value of RQ recorded during the post meal period. Energy expended with activity was calculated from the difference between total and resting metabolism.

### Fecal fat excretion

The amount of fat in dried feces (n = 7 cages of 5 mice in each group) was measured by the Laboratory of Functional Coprology, Pitié-Salpétrière Hospital, Paris France.

### Culture of explants from adipose tissue

Explants of epididymal adipose tissue were prepared from *ob/ob* male mice, following overnight fasting. Epididymal adipose tissue samples were cut into small pieces (1 mm^3^) under sterile conditions and rinsed in Dulbecco's modified Eagle medium (DMEM, Invitrogen). Explants (200 mg) were further incubated for 48 hours at 37°C in DMEM supplemented with 12.5 mM glucose, 200 IU/ml penicillin, 50 mg/l streptomycin, and 2% BSA, and further exposed to 1 µM JWH-133 (Tocris, France) or vehicle (DMSO 0.02%). After 48 hours, samples were frozen in liquid nitrogen and stored at −80°C before RNA extraction.

### Serum analysis

Blood was collected at the time of sacrifice, fasting glycemia were determined by Accu-Check active bands (Roche Diagnostics) and insulinemia by Elisa (Ultrasensitive mouse sensitive Elisa, Mercodia), respectively.

### Hepatic triglyceride quantification

Hepatic triglycerides were extracted from 50 mg of liver homogenates by homogenization in 1 ml of chloroform-methanol (2∶1 v/v) using TissueLyser (Qiagen). The homogenate was centrifuged for 10 min at 1000 g, and the lipid phase was diluted with 0.2 ml water. Following an additional centrifugation for 20 min at 2400 rpm, the lipid extract was collected from the lower phase, evaporated and dissolved in 1 ml of 2-propanol. Triglycerides were quantified with a triglyceride determination kit (Sigma), on liver samples from 10–15 animals per group.

### Insulin tolerance test

Mice (7–10 per group) underwent a 0.75 U/kg i.p. injection of human insulin (Insulin NPH; Lilly), following a 3 h fasting. Blood samples were taken from the tail vein for determination of glycemia at time 0 and 20, 30, 40, 60, 90, 120, and 180 min after the insulin injection.

### Hyperinsulinemic-euglycemic clamp

Hyperinsulinemic- euglycemic clamp was performed as previously described [22]. Briefly, 6h-fasted WT (n = 6) and *Cnr2* −/− (n = 7) mice fed a HFD for 15 weeks, received simultaneous infusions of insulin (18 mU.kg^−1^.min^−1^ for 3 h), and D-(^3^H)3-glucose (30 µCi.kg^−1^.min^−1)^, Perkin Elmer, Boston, MA) to ensure a detectable D-(^3^H)3-glucose enrichment. Glycemia was assessed throughout the infusion from blood samples collected from the tip of the tail vein and euglycemia was maintained by adjusting a variable infusion of 30% (wt/vol) glucose. Plasma glucose concentrations and D-(^3^H)3-glucose specific activity were determined in 5 µl of blood sampled from the tip of the tail vein every 10 min during the last hour of the infusion. The enrichment in D-(^3^H)3-glucose was determined from total blood after deproteinization by a Zn(OH)_2_ precipitation as previously described [22]. An aliquot of the protein-free supernatant was evaporated to dryness and mixed with scintillation fluid to determine the radioactivity corresponding to D-[^3^H]3-glucose. In a second aliquot of the same supernatant, glucose concentration was measured by the glucose oxidase method (Sigma).

### RNA preparation and RT-PCR

Total RNA was extracted from mice epididymal fat, liver and brain using RNeasy® Lipid Tissue Mini kit (Qiagen). Quantitative RT-PCR was carried out on a Light Cycler (Roche Diagnostics), as previously described [23]. Oligonucleotide primer sequences of the mouse genes studied were the following. mouse *Rn18S*: **sense** 5′-ACCAGAGCGAAAGCATTTGCCA-3′, **antisense**: 5′-ATCGCCAGTCGGCATCGTTTAT-3′; mouse *Cnr2*: **sense** 5′-GGATACAGAATAGCCAGGAC-3′, **antisense** 5′-GGAGCCGTTGGTCACTTCTG-3′; mouse *Cnr1*: **sense** 5′-GGGCAAATTTCCTTGTAGCA-3′, **antisense** 5′-CTGCAAGGCCGTCTAAGATCGACT-3′; mouse *Ccl2*: **sense** 5′-GGGCCTGCTGTTCACAGTT-3′, **antisense** 5′-CCAGCCTACTCATTGGGAT-3′; mouse *Emr1*: **sense** 5′-CTTTGGCTATGGGCTTCCAGTC-3′, **antisense** 5′-GCAAGGAGGACAGAGTTTATCGTG-3′; mouse *Tnf*: **sense** 5′-AATGGCCTCCCTCTCATCAGTT-3′, **antisense** 5′-CCACTTGGTGGTTTGCTACGA-3′. PCR amplified products were analyzed on a 2% agarose gel and sequenced.

### Immunohistochemistry

Immunohistochemical detection of F4/80 was performed in epididymal adipose formalin-fixed, parraffin-embedded tissue sections (8 µm), using non-diluted antibody to F4/80 (Serotec MCA 497) and biotinylated secondary antibody (1/50, Serotec AAR10B). The signal was amplified with phosphatase alkaline–conjugated streptavidin (1/20, Serotec), and alkaline phosphatase activity was revealed using fast red substrate–chromogen system (Dakocytomation). The total number of F4/80 expressing cells was quantified on at least 500 cells from 5 random fields in 7 vehicle-treated or 7 AM-630-treated WT mice fed a HFD, and 5 *Cnr2* −/− HFD-fed animals, using Image J software. Results are expressed as F4/80 stained cells/total cells.

### Statistics

Results are expressed as mean±SEM and were analyzed by either Mann and Whitney test, one or two way ANOVA as appropriate, using PRISM 4.0 software. *P*<0.05 was taken as the minimum level of significance.

## Results

### Characterization of CB2 receptor expression in the adipose tissue and the liver during obesity

**Figure 1:**
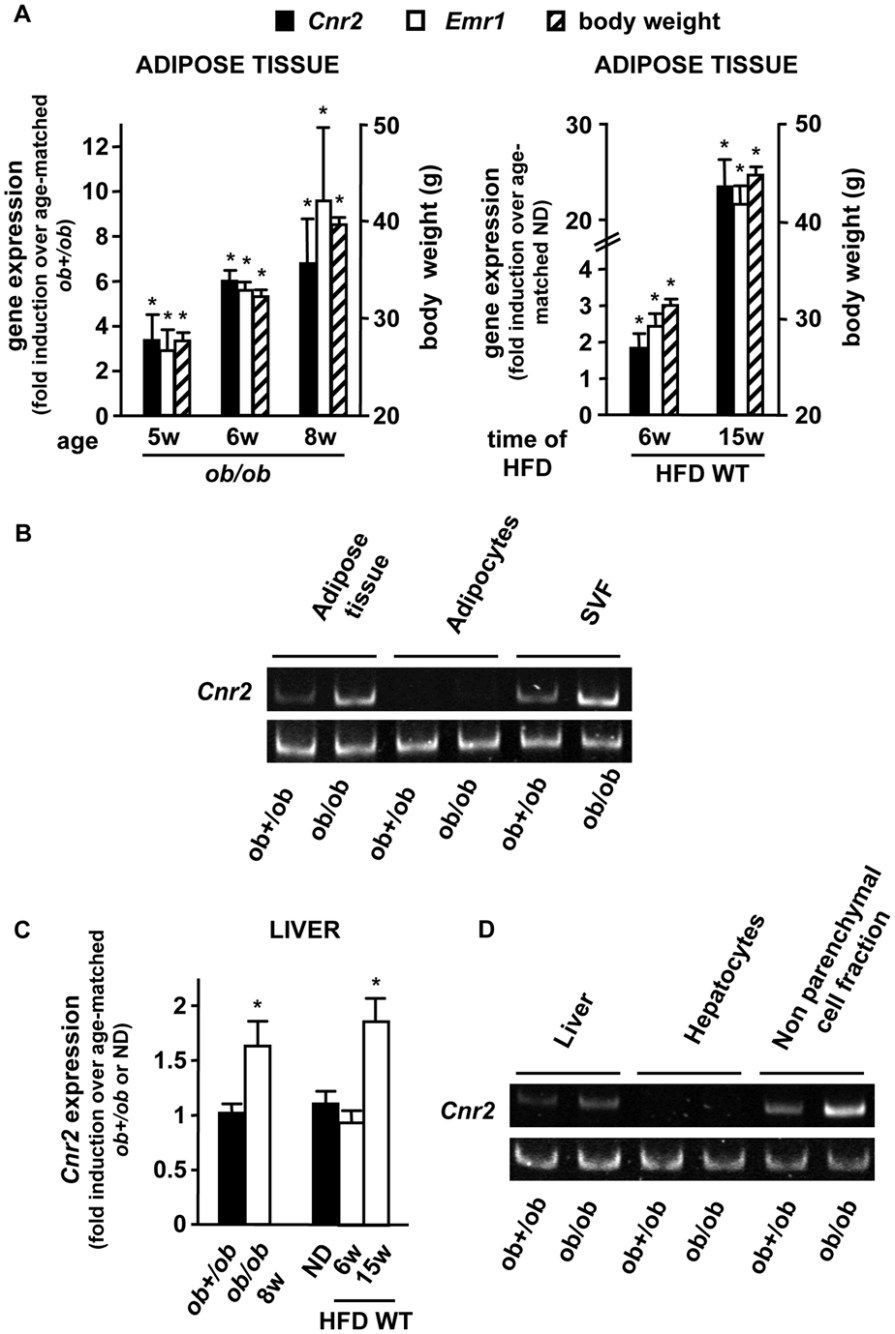
Selective distribution of *Cnr2* in the adipose tissue and regulation during obesity. A, Time course of *Cnr2* and *Emr1* expression in the epididymal adipose tissue of obese *ob/ob* and HFD-fed WT mice and corresponding body weight. B, *Cnr2* expression was quantified in the SVF and adipocyte fraction of *ob/ob* and *ob+/ob* mice. C, Time course of *Cnr2* expression in the liver of obese *ob/ob* and HFD-fed WT mice. D, *Cnr2* expression was quantified in the non parenchymal and parenchymal liver cell fractions of *ob/ob* and *ob+/ob* mice. *p<0.05 for *ob/ob vs* age-matched *ob+/ob* mice or for HFD-fed *vs* age-matched ND-fed mice.

We first characterized the expression of *Cnr2* (encoding CB2) in the adipose tissue and in the liver, and its regulation overtime during obesity, using genetically obese leptin-deficient *ob/ob* mice and wild type (WT) C57Bl/6J mice fed a high fat diet (HFD) for 6 or 15 weeks. In the two models, *Cnr2* expression was detected in the epididymal adipose tissue of lean animals, and its expression was enhanced in obese mice in parallel to body weight increase (**Fig 1A**). Moreover, in both models, the induction of fat *Cnr2* expression was correlated with adipose tissue inflammation, as shown by the concurrent increase in the expression of *Emr1*, encoding the macrophage related marker F4/80 (**Fig 1A**). In keeping, epididymal adipose tissue fractionation experiments showed that *Cnr2* was predominantly expressed in the stromal vascular fraction, both in lean *ob+/ob* and obese *ob/ob* animals, and was barely detectable in the adipocyte fraction (**Fig 1B**). Obesity was associated with a modest 1.8-fold increase in *Cnr2* expression in the liver, both in *ob/ob* and HFD-fed animals (**Fig 1C**). In keeping with findings in the adipose tissue, *Cnr2* was primarily expressed by the non parenchymal liver cell fraction in lean *ob+/ob* and in obese *ob/ob* animals, whereas *Cnr2* was hardly detectable in the hepatocyte fraction (**Fig 1D**). Overall, these results indicate that obesity enhances *Cnr2* expression in the non parenchymal cell fraction of adipose tissue and liver.

### CB2 receptors promote obesity-associated inflammation

**Figure 2:**
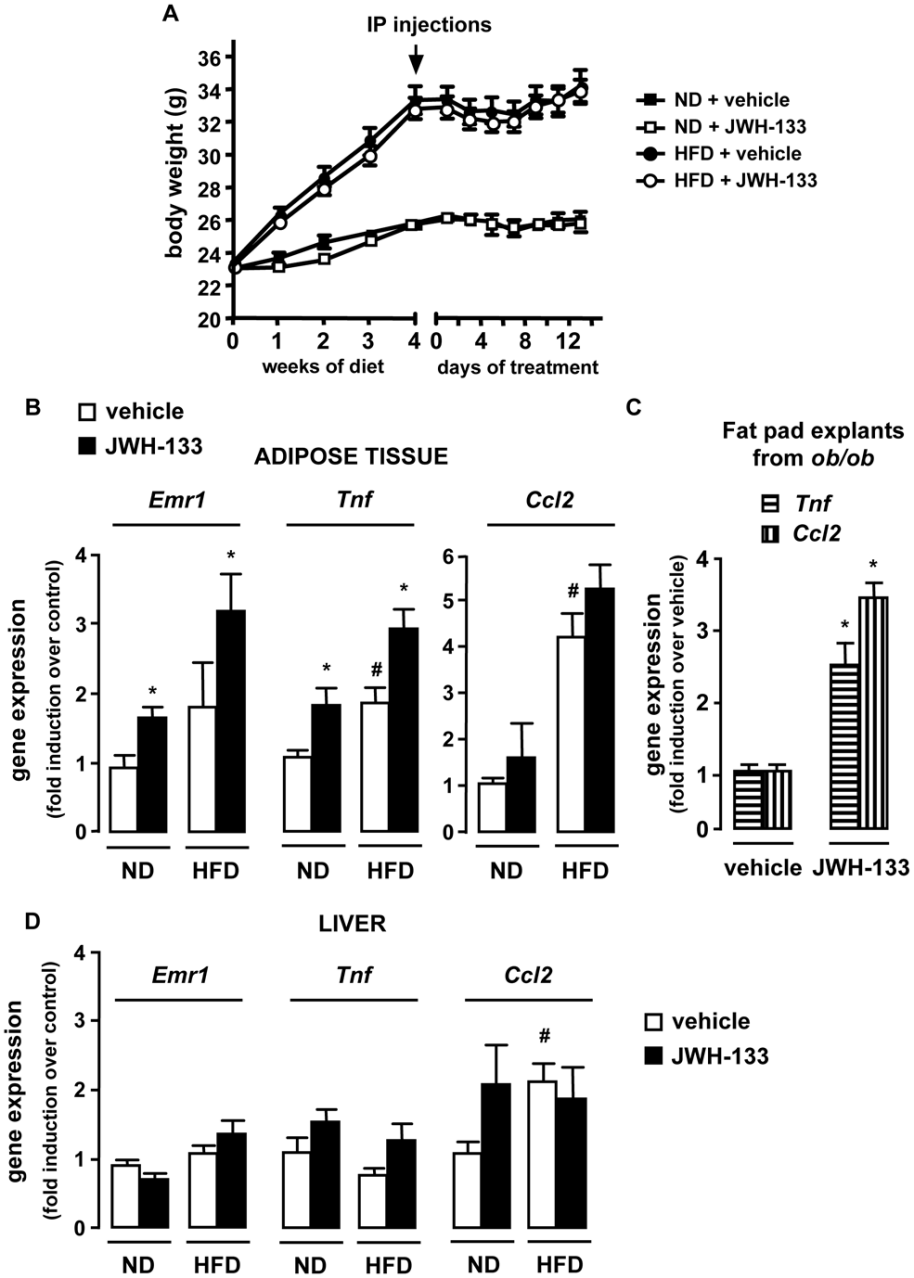
Activation of CB2 receptors by JWH-133 enhances fat inflammation in HFD-fed mice. WT mice were fed a HFD or a ND for 6 weeks, and treated with JWH-133 (3 mg/kg) or vehicle i.p daily during the last 15 days. A, Body weight progression during JWH-133 treatment. B, JWH-133 increases adipose tissue inflammation. Fold induction of adipose tissue *Emr1*, *Tnf* and *Ccl2* expression over control conditions (ND-fed mice injected with vehicle). C, Activation of CB2 receptors with 1 µM JWH-133 for 48 hours up-regulates *Tnf and Ccl2* expression in explants isolated from *ob/ob* mice fat pads. D, Impact of JWH-133 on hepatic inflammation. Fold induction of hepatic *Emr1*, *Tnf* and *Ccl2* expression over control conditions (ND-fed mice injected with vehicle). *p<0.05 for JWH-133 *vs* vehicle. #p<0.05 for HFD-fed *vs* ND-fed mice.

Given the regulatory role of *Cnr2* in inflammation [15], we next explored the possibility that CB2 receptors may regulate fat and liver inflammation. To that aim, C57Bl/6J mice were fed a HFD or a normal diet (ND) and sacrificed after 6 weeks, i.e at the early onset of inflammation in obese mice. In addition, both groups of mice received a daily intraperitoneal injection of the selective CB2 receptor agonist JWH-133 (3 mg/kg) or its vehicle during the last 2 weeks [24], [25]. Administration of JWH-133 did not affect body weight progression of either HFD or ND-fed mice (**Fig 2A**). As expected, vehicle-treated HFD-fed mice showed a moderate induction of epididymal fat *Emr1* and *Tnf* expressions, whereas the expression of *Ccl2*, encoding MCP-1, was already strongly increased (**Fig 2B**). Interestingly, administration of JWH-133 to ND-fed WT mice induced *Emr1* and *Tnf* expression, as compared to vehicle-treated ND counterparts (**Fig 2B**). Moreover, the compound further potentiated inductions of fat *Emr1* and *Tnf* in HFD-fed animals (**Fig 2B**). However, JWH-133 treatment had no effect on *Ccl2* expression either in ND or in HFD-fed mice (**Fig 2B**). The impact of JWH-133 was also investigated in cultured fat pads isolated from *ob/ob* mice. Cultured fat pads exposed to JWH-133 showed a marked induction in both *Tnf* and *Ccl2* expressions (**Fig 2C**). Finally, in the liver of mice fed a ND or a HFD, JWH-133 had little or no effect on *Ccl2* and *Tnf* expression respectively (**Fig 2D**). Taken together, these data demonstrate that the role of CB2 receptor is critical in adipose tissue inflammation, but less determinant in liver inflammation.

**Figure 3:**
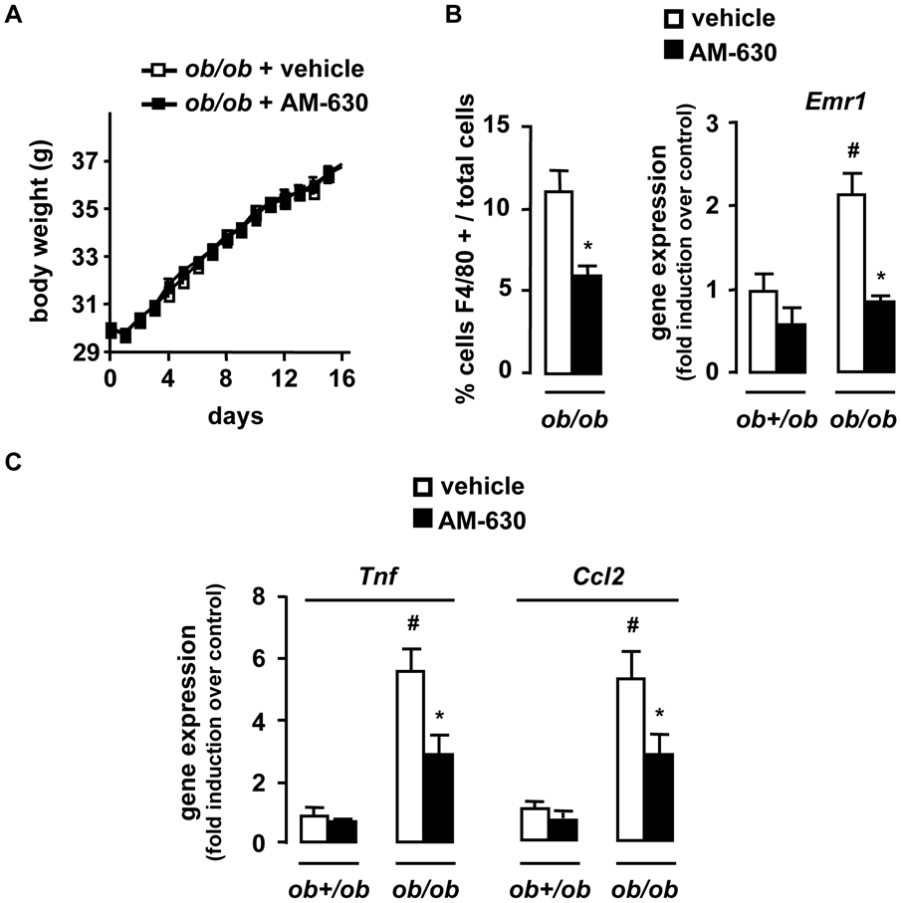
Pharmacological inactivation of CB2 receptors reduces the inflammatory response in the adipose tissue. *ob/ob* and *ob+/ob* mice were daily treated for 15 days with an intraperitoneal injection of AM-630 (1 mg/kg) or vehicle. A, Body weight progression of AM-630 and vehicle-treated *ob/ob* mice over time. B, C AM-630 reduces adipose tissue inflammation, as assessed by quantification of fat F4/80 stained cells/total cells and fold induction of fat *Emr1*, *Tnf* and *Ccl2* expression over control conditions (*ob+/ob* mice injected with vehicle). *p<0.05 for AM-630 *vs* vehicle. #p<0.05 for *ob/ob vs ob+/ob* mice.

**Figure 4:**
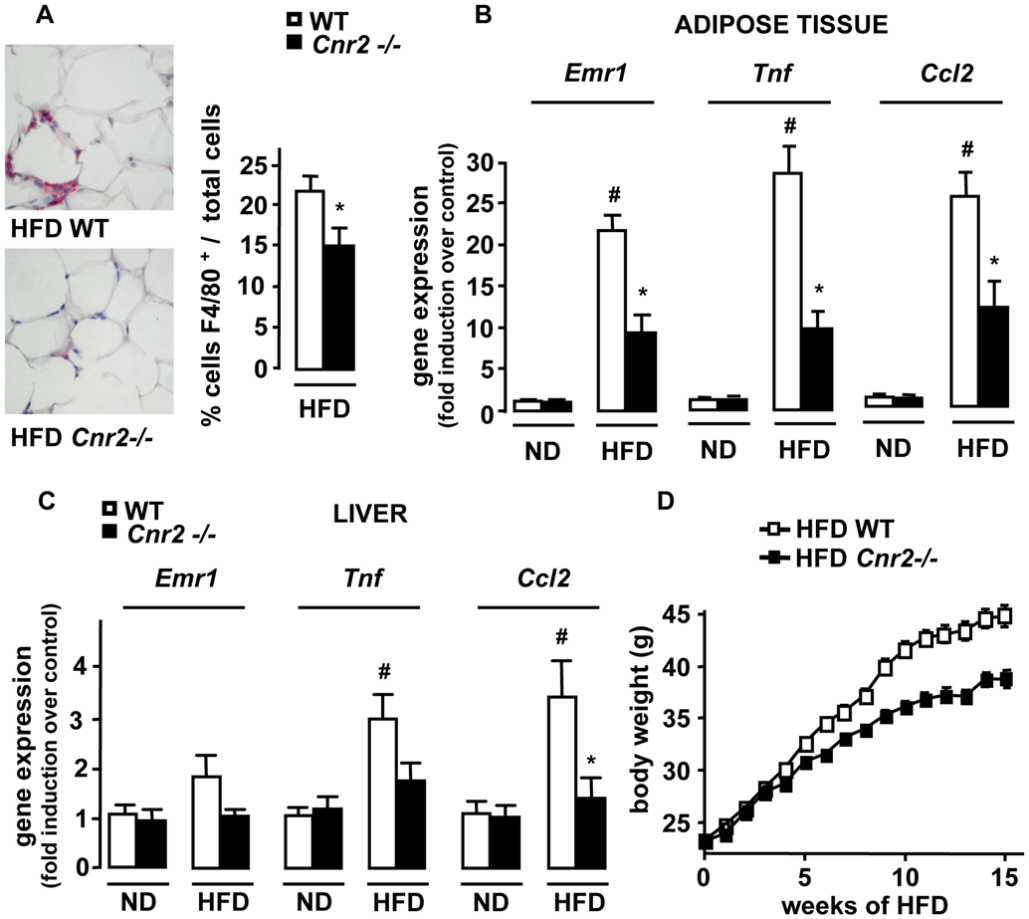
*Cnr2* knock-out reduces the inflammatory response in the adipose tissue and the liver. WT and *Cnr2* −/− mice were fed a HFD or a ND for 15 weeks. A, Macrophage infiltration into epididymal fat was assessed by immunohistochemical detection of F4/80 (magnification ×400) and quantification of F4/80 stained cells/total cells. B, Fold induction of fat *Emr1*, *Ccl2* and *Tnf* expression over control conditions (ND-fed WT mice). C, Fold induction of hepatic *Emr1*, *Ccl2* and *Tnf* expression over control conditions (ND-fed WT mice). D, Body weight progression over time of WT and *Cnr2* −/− mice fed a HFD for 15 weeks (p<0.05 by two way ANOVA). *p<0.05 for HFD-fed *Cnr2* −/− *vs* HFD-fed WT mice. #p<0.05 in HFD-fed WT *vs* ND-fed WT mice.

We next investigated whether CB2 receptor antagonism reduced obesity-associated inflammation. A 2-week treatment of *ob/ob* mice with the CB2 receptor antagonist AM-630 (1 mg/kg/day) did not affect body weight progression [26] (**Fig 3A**) while reducing adipose tissue inflammation, as reflected by the reduction in the accumulation of F4/80 positive cells and of *Emr1*, *Tnf* and *Ccl2* expression (**Fig 3B and C**). Moreover, in control lean *ob+/ob* mice there was a trend towards reduced fat inflammation in AM-630 treated animals compared to lean vehicle-exposed controls (**Fig 3B and C**). These results were confirmed in *Cnr2−/−* mice fed a HFD for 15 weeks. Compared to HFD-fed WT mice, HFD-fed *Cnr2−/−* animals exhibited a significant reduction in fat macrophage density (**Fig 4A**) and a lesser induction of *Emr1*, *Tnf* and *Ccl2* (**Fig 4B**). Finally, livers of HFD-fed *Cnr2−/−* mice also showed a decline in inflammation, as compared to their WT counterparts (**Fig 4C**). Overall, these data demonstrate that CB2 receptor inactivation reduces inflammation both in adipose tissue and in the liver of obese animals.

### Deletion of *Cnr2* reduces body weight progression under HFD

Surprisingly, *Cnr2* knock-out was also associated with a slower body weight progression that becomes significant after 8 weeks of HFD (p<0.05 by two way ANOVA) (**Fig 4D**). Accordingly, *Cnr2 −/−* mice also displayed smaller adiposity index and adipocyte size compared to WT animals (**Table 1**), and reduced circulating leptin levels, whereas serum adiponectin levels were unchanged (**Table 1**). Subsequent experiments investigated mechanisms underlying *Cnr2* impact on obesity.

**Table 1:** Metabolic parameters of HFD WT and *Cnr2* −/− mice.

	HFD WT	HFD *Cnr2* −/−
*Metabolism*		
Food intake (g/day)	2.6±0.00	2.7±0.1
Adiposity index (% of body weight)	9.6±0.4	6.8±0.8**
Adipocyte size (µm^2^)	9,937±391	6,166±1,009*
Metabolic rate (W)		
Basal	0.31±0.02	0.30±0.00
Total	0.44±0.02	0.42±0.02
Spontaneous locomotor activity (kJ/g)	0.14±0.01	0.14±0.00
RQ (peak post meal)	0.92±0.02	0.85±0.01*
Fecal lipid (% of dry fecal weight)	19.9±1.5	25.9±3.1*
*Cnr1 expression*		
Brain	1.02±0.01	1.42±0.30
Liver	1.21±0.33	0.43±0.06*
Adipose tissue	1.04±0.07	0.49±0.08***
*Adipokines*		
Leptin (µg/L)	61±7	19±5***
Adiponectin (mg/L)	11.5±0.6	11.2±0.18

* p<0.05 *vs* HFD WT.

** p<0.01 *vs* HFD WT.

*** p<0.001 *vs* HFD WT.

Since the cannabinoid receptor CB1 plays a key role in the pathogenesis of obesity by increasing food intake and reducing energy expenditure, we first characterized *Cnr1* expression in brain, adipose tissue and liver. There was no difference in brain *Cnr1* expression between HFD-fed WT and *Cnr2−*/− mice. However, surprisingly, *Cnr1* expression was decreased in the adipose tissue and the liver of HFD-fed *Cnr2 −/−* mice as compared to their WT counterparts (**Table 1**). In contrast, livers and adipose tissue isolated from JWH-133 or AM-630 treated animals showed no difference in *Cnr1* levels (not shown). These data suggested that the reduction of *Cnr1* expression in the adipose tissue and liver observed in *Cnr2 −/−* mice might account for the reduction in body weight progression in these animals, a reduction that is not observed in WT mice treated with pharmacological agonists or antagonists of CB2 receptors. The next series of experiments were therefore designed to further delineate the mechanisms underlying the *Cnr2−/−* mice phenotype.

Several studies have reported that *Cnr1* knock-out decreases food intake and increases energy expenditure. However, food intake was similar in *Cnr2−*/− and WT mice fed a HFD (**Table 1**). Indirect calorimetry showed that HFD-fed *Cnr2 −/−* and WT mice had similar basal and total metabolic rate, exhibited the same level of spontaneous activity (**Table 1**), and equivalent thermogenic response to feeding (not shown). Nevertheless, *Cnr2−/−* mice tended to display a lower pre-meal, post meal and overall respiratory quotient, although the differences with WT animals did not reach statistical significance (data not shown). In addition, the peak of meal-induced RQ increase was significantly reduced in *Cnr2 −/−* animals, compared to WT (**Table 1**), therefore indicating that *Cnr2* knock-out may reduce the inhibition of lipid oxidation induced by feeding. Finally, quantification of fat elimination in faeces showed a modest albeit significant increase in fecal lipid output in HFD-fed *Cnr2 −/−* mice, as compared to WT counterparts **(** **Table 1** **)**, by a mechanism that may involve inhibition of pancreatic lipase gene expression (Cadoudal et al, preliminary results). Overall, these data indicate *Cnr2* knock-out attenuates the progression of obesity by a mechanism distinct from that elicited by CB1 antagonism, possibly by increasing both lipid oxidation and fecal fat excretion.

### CB2 receptors promote insulin resistance

**Figure 5:**
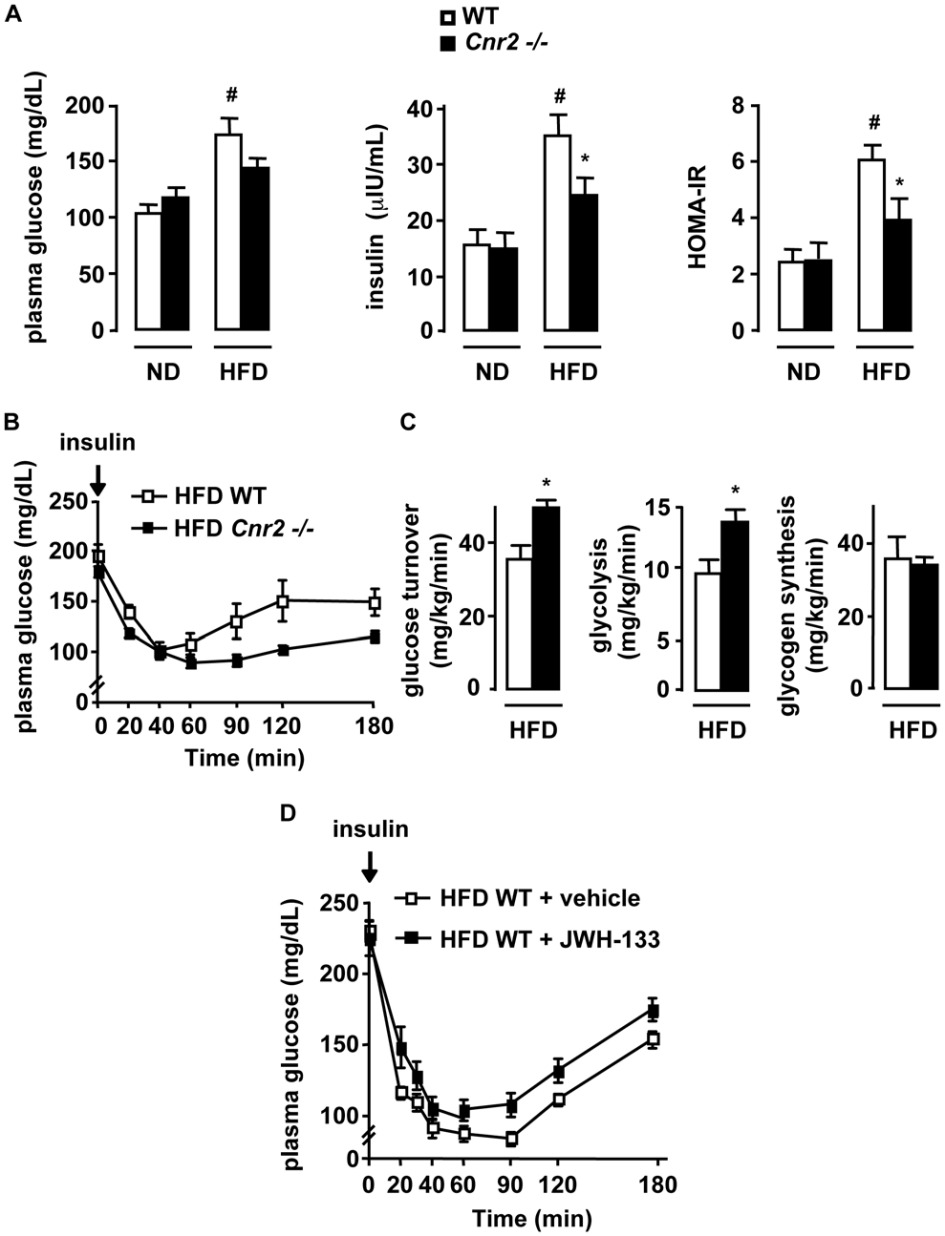
CB2 receptors potentiate insulin resistance in HFD-fed mice. A–C, *Cnr2* knock-out reduces obesity-induced insulin resistance. WT and *Cnr2* −/− mice were fed a ND or a HFD for 15 weeks A, Fasting glycemia, insulinemia, HOMA-IR; B, Blood glucose level during insulin tolerance tests in HFD-fed mice (p<0.05 by two way ANOVA). C, Hyperinsulinemic-euglycemic clamp in HFD-fed mice, as assessed by glucose turnover rate, whole body glycolysis and glycogen synthesis, following insulin stimulation. D, CB2 receptor activation increases insulin resistance. Blood glucose level during insulin tolerance tests in WT mice fed a HFD for 6 weeks and treated daily with an i.p injection of JWH-133 (3 mg/kg) or vehicle during the last 15 days of HFD (p<0.05 by two way ANOVA). * p<0.05 for HFD-fed *Cnr2* −/− *vs* HFD-fed WT mice. # p<0.05 in HFD-fed WT *vs* ND-fed WT mice.

We next examined the impact of CB2 receptors on obesity-associated insulin resistance, a reported consequence of inflammation. As expected, WT mice fed a HFD for 15 weeks developed hyperglycemia and hyperinsulinemia, resulting in increased HOMA-IR (**Fig 5A**). In contrast, the increase in insulin plasma levels and HOMA-IR was significantly lower in HFD-fed *Cnr2 −/−* mice **(** **Fig 5A**). Consistent with these results, insulin tolerance test showed that HFD-fed *Cnr2 −/−* mice were more insulin sensitive than their WT counterparts (p<0.05 by two way ANOVA) (**Fig 5B**). These findings were further corroborated in hyperinsulinemic-euglycemic clamp experiments **(** **Fig 5C** **)**. Indeed, glucose turnover and glycolysis rate were increased in HFD-fed *Cnr2 −/−* mice, whereas glycogen synthesis rate remained unchanged, confirming the insulin sensitizing effects of *Cnr2* knock-out **(** **Fig 5C**). Accordingly, in mice exposed to a HFD for 6 weeks, treatment with the CB2 agonist JWH-133 enhanced insulin resistance, as assessed by the insulin tolerance test (p<0.05 by two way ANOVA) **(** **Fig 5** **D)**. Collectively, these results demonstrate that CB2 receptors enhance obesity-associated insulin resistance.

### CB2 receptors promote fatty liver

**Figure 6:**
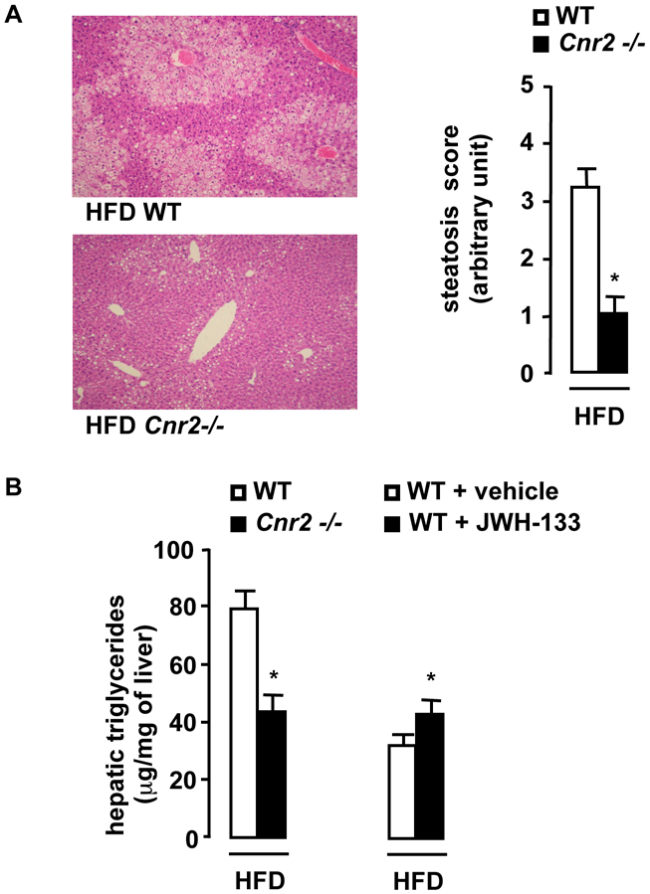
CB2 receptors promote the development of hepatic steatosis. A, *Cnr2* knock-out blunts steatosis. Representative liver tissue sections (magnification ×100), mean steatosis score and hepatic triglycerides in WT and *Cnr2 −/−* mice fed a HFD for 15 weeks. B, Hepatic triglycerides in HFD-fed WT or *Cnr2−/−* mice for 15 weeks and in vehicle or JWH-133-treated (3 mg/kg) HFD-fed WT mice for 6 weeks. *p<0.05 for JWH-133 *vs* vehicle or for HFD-fed *Cnr2 −/− vs* HFD-fed WT mice.

Obesity-associated fatty liver has been causally linked to adipose tissue inflammation and insulin resistance. As expected, WT mice fed a HFD for 15 weeks developed severe fatty liver **(** **Fig 6A** **)**. In contrast, HFD-fed *Cnr2 −/−* animals exhibited minimal steatosis, as illustrated by the decrease in the steatosis score **(** **Fig 6A** **)** and the decline in liver triglyceride concentration, compared to HFD-fed WT animals **(** **Fig 6B** **)**. In keeping with these results, administration of JWH-133 enhanced liver triglyceride accumulation in WT mice fed a HFD for 6 weeks **(** **Fig 6B** **)**. Taken together, these findings indicate that CB2 receptors potentiate obesity-associated fatty liver.

## Discussion

Our study unravels a previously unrecognized contribution of CB2 receptors to the pathogenesis of obesity associated-comorbodities *via* an impact on obesity-associated inflammation, insulin resistance and fatty liver.

Recent studies have conclusively shown that adipose tissue and liver inflammation plays a major role in obesity-associated insulin resistance and NAFLD [4]–[7], [27], [28]. Our data indicate that during obesity progression, *Cnr2* expression undergoes a strong increase in adipose tissue parallel to the development of fat inflammation, and a moderate induction in the liver. Tissue fractionation experiments show that enhancement of *Cnr2* expression arises from the macrophage-enriched stromal vascular fraction of the adipose tissue and the non parenchymal liver cell fraction, whereas *Cnr2* expression in adipocytes and hepatocytes remains negligible. Pharmacological activation of CB2 receptors has no significant impact on liver inflammation but promotes a significant enhancement of adipose tissue inflammation at an early stage. Indeed, administration of JWH-133 at the onset of inflammation, i.e. in mice fed a HFD for 6 weeks, increases fat macrophage infiltration and the related inflammatory response. Moreover, pharmacological and genetic CB2 receptor inactivation reduce adipose tissue and liver inflammation in the long term, as shown in *ob/ob* mice or animals fed a HFD for 15 weeks. Interestingly, in isolated fat pads, the CB2 receptor agonist JWH-133 upregulates *Ccl2* and *Tnf*, demonstrating the direct impact of CB2 receptors to adipose tissue inflammation. Nevertheless, in light of previous data showing *Cnr2* expression in human adipocytes and in adipocyte cell lines [29], [30], the contribution of adipocyte CB2 receptors to fat inflammation cannot be definitively ruled out. Mechanisms underlying the impact of CB2 receptors on macrophage recruitment remain to be determined. Potential pathways could involve enhanced chemotaxis and/or increased macrophage migration, considering reported stimulatory effects of CB2 receptors on these two functions in other conditions [15], [31]–[33]. The relative contribution of liver *vs* adipose tissue inflammation remains to be determined. Nevertheless, our data unravel the critical role of CB2 receptors in obesity-associated inflammation.

Insulin resistance associated with obesity has been linked to adipose tissue inflammation [4]–[7]. In keeping with these studies, our data suggest that proinflammatory properties of adipose tissue CB2 receptors may contribute to the development of insulin resistance. Indeed, under HFD, JWH-133-treated WT mice show enhanced insulin resistance and fat inflammation, whereas *Cnr2 −/−* mice display improved insulin sensitivity and reduced fat inflammation. It should also be stressed that, aside from their impact on fat inflammation, CB2 receptors expressed by other insulin sensitive tissues, including skeletal muscle and liver [23], [34], [35] may also contribute to insulin resistance. In addition, deleterious effects of CB2 receptors on insulin secretion may also be expected, in light of the inhibition of insulin secretion triggered by CB2 receptors expressed in pancreatic β cells [35], [36].

Our results indicate that fatty liver, a hallmark of obesity-associated metabolic syndrome, is enhanced by the CB2 receptor agonist JWH-133 and blunted by *Cnr2* knock-out. Recent data have conclusively shown a causal relationship between obesity-induced overproduction of fat inflammatory mediators such as *Ccl2*, insulin resistance and steatogenesis. Indeed, insulin resistance enhances the release of non-esterified fatty acids from adipose tissue, leading to increased fatty acid storage in hepatocytes [10]. Moreover, mice overexpressing *Ccl2* specifically in adipose tissue exhibit insulin resistance and increased hepatic triglyceride content. Conversely, knock-out of *Ccl2* or its receptor *Ccr2*, improves insulin sensitivity and prevents steatosis [27], [28], [37]. Therefore, it is tempting to speculate that CB2 receptors may enhance fatty liver at least partly by increasing adipose tissue inflammation and the related induction of *Ccl2*. Other steatogenic mechanisms may include enhancement of lipogenesis and/or inhibition of fatty acid β-oxidation in hepatocytes, following direct activation of *Cnr2*. However, although expressed in the liver [23], CB2 receptors are undetectable in steatotic hepatocytes.

Recent studies in obese patients have reported elevated circulating levels of the endogenous CB2 ligand 2-arachidonoylglycerol [38] and increased production of the compound by fat cells [39]. These observations, together with our findings demonstrating induction of CB2 receptor in adipose tissue and liver, indicate that alike CB1 tone, the endogenous CB2 tone is increased during obesity [40], [41]. Clinical and experimental studies in obesity have also linked overactivation of CB1 receptors to body weight progression, insulin resistance and fatty liver [11], [14], [42]–[45]. Interestingly, although both receptors display convergent effects, pathways underlying CB1 effects are markedly different from those affected by CB2. Indeed, CB1 receptors mainly enhance food intake, reduce peripheral energy expenditure and enhance hepatic lipogenesis *via* CB1 receptors expressed by hepatocytes. In contrast, pharmacological modulation of CB2 receptors has no impact on either food intake or body weight progression and predominantly affects adipose tissue inflammation. It should however be pointed out that *Cnr2* knock-out was associated with a slight reduction of body weight progression under HFD, that was not observed in WT animals exposed to CB2 agonists or antagonists. This discrepancy may result from developmental defects linked to lifelong absence of CB2 receptors or may be due to the short duration of treatment with the CB2 targeting molecules in our experiments. Nevertheless, our findings indicate that distinct CB1 and CB2 receptor-dependent pathways significantly contribute to the pathogenesis of insulin resistance and NAFLD.

In conclusion, the present study uncovers the potent proinflammatory properties of CB2 receptors during experimental obesity and shows the deleterious impact of CB2 receptors on insulin resistance and fatty liver. These findings further document the crucial role of the endocannabinoid system in metabolic comorbidities associated to obesity, and unravel the previously unrecognized role of CB2 receptors. They also demonstrate that both CB1 and CB2 receptors display convergent effects, *via* complementary pathways [11], [13], [14]. Finally, our results add novel evidences underscoring the role of CB2 receptors in the pathogenesis of inflammatory disorders [15] and liver diseases [12], [17], [23], [45]–[48]. Lastly, our results suggest that CB2 receptor antagonism may open a novel therapeutic approach for the management of insulin resistance and fatty liver.
